# Appropriate complementary feeding practices and associated factors among mothers of children age 6–23 months in Southern Ethiopia, 2015

**DOI:** 10.1186/s12887-016-0675-x

**Published:** 2016-08-19

**Authors:** Tigist Kassa, Berhan Meshesha, Yusuf Haji, Jemal Ebrahim

**Affiliations:** 1 Affiliate of School of Public & Environmental Health, College of Medicine and Health Science, Hawassa University, Hawassa, Ethiopia; 2 School of Public & Environmental Health, College of Medicine and Health Science, Hawassa University, Hawassa, Ethiopia

**Keywords:** Appropriate complementary feeding, Associated factors, Children, Ethiopia

## Abstract

**Background:**

Poor complementary feeding of children aged 6–23 months contributes to the characteristics negative growth trends and deaths observed in developing countries. Evidences have shown that promotion of appropriate complementary feeding practices reduces the incidence of stunting and leads to better health and growth outcome. This study was aimed at assessing practices of complementary feeding and associated factors among mothers of children aged 6–23 months.

**Methods:**

A community-based cross sectional study design was conducted among 611 mothers who had children with 6–23 months of age in the ten randomly selected Kebeles (smallest administrative unit). A multistage sampling technique was used to identify study subjects. Data were collected using pre-tested structured questionnaire. Data were entered in to Epi info version 3.5.1. Data cleaning and analysis were done using SPSS version 16. Odds ratios (ORs) with 95 % confidence interval (CI) were computed to measure the strength of association.

**Results:**

The response rate was 97.6 % (611/626). The practices of timely initiation of complementary feeding, minimum meal frequency and minimum dietary diversity were 72.5, 67.3 and 18.8 % among mothers of 6–23 months aged children, respectively. The practice of appropriate complementary feeding was 9.5 %. Child’s age (12–17 and 18–23 months) [Adjusted OR: 2.75 (95 % CI: 1.07 7.03), 2.64 (95 % CI: 1.06 6.74)], educational level of mother (primary and secondary and above schools) [AOR: 3.24 (1.28 8.20), 3.21 (1.1.07 9.70)], and smaller family size [AOR: 12.10 (95 % CI: 1.10 139.7)] were found to be independent predictors of appropriate complementary feeding practice of 6–23 months old children.

**Conclusion:**

Low appropriate complementary feeding of children aged 6–23 months was observed. Mothers who are illiterate, children age 6–11 months and families with large size were associated factors for inappropriate feeding practices. Therefore, nutritional counseling on child feeding practices were recommended.

## Background

Malnutrition remains a major health problem that in 2011, globally, 165 million children younger than 5 years were stunted, more than 100 million were underweight and 52 million were wasted [1]. A recent analysis of Demographic and Health surveys (DHS) data from twenty-one countries revealed that poor complementary feeding of children aged 6–23 months contributes to the characteristics negative growth trends observed in developing countries [2]. In sub-Saharan African Regions, for example, suboptimal infant feeding practices, poor quality of complementary foods, micronutrient deficiencies and frequent infections have mainly contributed to the high mortality among infants and young children [3].

Similarly, malnutrition is a significant health problem for infants and young children in Ethiopia. The Ethiopian DHS, 2011 show that national rates of stunting, underweight and wasting among under 5 years children were 44.4, 28.7, and 9.7 % respectively, and have declined only slightly in the past 5 years [4, 5].

Understanding the effect of infant and young child feeding (IYCF) practices on improving the nutritional status of children under two years of age, the World Health Organization (WHO) developed a set of core indicators to assess IYCF practices [6]. These indicators incorporated both breast-feeding and complementary feeding linked practices. Appropriate feeding practices, therefore, include timely initiation of feeding of solid and semi-solid foods from age 6 months and to improve the quantity and quality of foods children consume, while maintaining breastfeeding [6, 7].

There is strong evidence that the promotion of appropriate complementary feeding practices reduces the incidence of stunting and leads to better health and growth outcome [1, 7]. Therefore, as an effective intervention strategy for malnutrition, WHO and United Nation for Child Fund (UNICEF) recommended introduction of adequate complementary foods at 6 months with continued breastfeeding for 2 years of age or beyond [6]. And this will have a potential to improve the nutritional status of children in developing countries [8]. However, in Ethiopia, the prevalence of appropriate complementary feeding practices among children aged 6–23 months was very low (4.8 %) [4].

Previous studies conducted elsewhere on factors associated with appropriate complementary feeding practices of children aged 6–23 months show higher maternal and paternal education, better household wealth, exposure to media, adequate antenatal and post-natal contacts, child’s sex and age, institutional delivery, low parity, maternal occupation, urban residence, knowledge & frequency of complementary feeding and receiving feeding advice in immunization as determinant factors for appropriate complementary feeding [9–25].

These evidences strongly call for the need to improvement of complementary feeding practices but there is a scarce evidences for overall complementary feeding practices and associated factors in the region, especially, in the study area. To improve complementary feeding practice through this essential time of growth and development of the child, assessment of complementary feeding practices and its factors are vital [6, 7, 26].

The current study was aimed at determining the prevalence of appropriate complementary feeding practices and its associated factors among children aged 6–23 months residing in the rural area of Southern Ethiopia. And the findings of this study will provide some critical insights for further research and interventions.

## Methods

### Study setting and sample

The community based cross sectional survey was conducted from February 10–25, 2015 in rural community of Arsi Negele Woreda (District). The woreda has a total population of 257,428 residing in 43 kebeles (smallest administrative unit) with 127,470 (49.5 %) male and 129,950 (50.5 %) female as projected for 2014 from 2007 national Census. Children aged 6–23 months of age in the district constituted 6 % (14,702) of the population. The livelihood of more than 65 % of the district population is based on farming. The main crops grown in the area are wheat, maize and teff (a species of Eragrostis native to Ethiopia). The source population was all mothers having children 6–23 months of age and residing in the study area. The study population was selected mothers with children aged 6–23 months and resided in the study area for more than 6 months. Sample size was determined using a single population proportion at 95 % CI level (Z (1-ά/2) = 1.96), 44.7 % prevalence of minimum meal frequency from previous survey [23], 5 % margin of error, and 5 % level of significance. Design effect for cluster surveys, *DEFF* of 1.5, was used as a multiplier to increase the sample size to account for the effect of the cluster sampling method. After considering 10 % non-responses and refusals, the total sample size required for the study was 626 of mothers with 6–23 months of age children.

### Sampling procedure

A multistage sampling technique was used to select the study subjects. Ten kebeles were randomly selected using simple random sampling method from 43 kebeles. The total population size in the ten selected kebeles was 52,934 of which 2941 was children 6–23 months of age. Proportional allocation of the calculated sample (626) was done among the selected kebeles. To get the individual sample units (subjects) at household level, a community health management information system (CHMIS) list (a documented list of all target group of the kebele) has been used from the health post to get list of target group. Using systematic random sampling a child was selected in each kebele and his/her mother was interviewed accordingly. From each household one eligible child with mother at the time of survey was selected and the process had been continuing until next **K** in the same direction.

### Methods of data collection

A pre-tested structured questionnaire initially developed in English and then back translated into local language (Afan Oromo) was used for data collection (feeding practices and 24 h dietary recall parts of the questionnaire was adopted from WHO/UNICEF tool). The main sections of the questionnaire comprised of socio-demographic characteristics, maternal health care related information, feeding practices, and dietary assessment using 24 h recall. Data were collected by ten diploma nurses after receiving an intensive training on the aim of the study, procedures to be followed, as well as approach of client during interview for 2 days. The technique for data collection was a face-to-face interview method and from each household one eligible child age 6–23 with mother at the time of survey had been selected. Appropriate supervision had been conducted by the investigators at each respective kebeles during data collection.

### Complementary feeding indicators

Complementary feeding practices were assessed using the key indicators recommended by the WHO/UNICEF in 2008 which include introduction of solid, semi-solid or soft foods, minimum dietary diversity, minimum meal frequency and minimum acceptable diet calculated for the age ranges 6–11, 12–17 and 18–23 months of age, and based on a 24-h recall of the child’s dietary intake. These indicators include:

- Timely introduction of solid, semi-solid or soft foods: the proportion of infants 6–23 months of age who starts complementary foods (solid, semi-solid or soft) at 6 month [6].
- Minimum dietary diversity: the proportion of children 6–23 months of age who receive foods from four or more food groups during the previous day. The seven food groups used for tabulation of this indicator were: grains, roots and tubers; legumes and nuts; dairy products (milk, yoghurt and cheese); flesh foods (meat, fish, poultry and liver/organ meats); eggs; vitamin A-rich fruits and vegetables; and other fruits and vegetables [6].
- Minimum meal frequency: the proportion of breastfed and non-breastfed children 6–23 months of age who receive solid, semi-solid or soft foods the minimum number of times or more (minimum is defined as: two times for breastfed infants 6–8 months; three times for breastfed children 9–23 months; and four times for non-breastfed children 6–23 months) in the previous day [6].
- Minimum acceptable diet: the proportion of breastfed children 6–12 months of age who had at least the minimum dietary diversity and the minimum meal frequency during the previous day, and non-breastfed children 6–23 months of age who received at least two milk feedings and had at least the minimum dietary diversity not including milk feeds and the minimum meal frequency during the previous day [6].

### Complementary feeding practices

Appropriate complementary feeding quantified using a composite indicator comprising three of the WHO core IYCF indicators that relate closely to complementary feeding. These are timely introduction of solid, complementary feeding, and minimum dietary diversity and minimum meal frequency. If a child fulfilled the above three criteria, we classified as having received appropriate complementary feeding.

### Data analysis

Before analyses, data were checked for completeness, inconsistencies and entered using Epi info version 3.5.1 statistical software. Then the data were exported to SPSS for windows version 16.0 (SPSS Inc. version 16.1, Chicago, Illinois) and coded, cleaned and analyzed. Descriptive statistics was used to show socio demographic characteristics and prevalence of complementary feeding practices. To verify the variables associated with appropriate complementary feeding practices, variables that show a *P*. value < 0.1 in the bivariate analyses were re-entered into multivariable logistic regression models to control for potential confounders. A *p* value < 0.05 was considered statistically significant. Adjusted Odds Ratios and their 95 % Confidence Intervals were reported.

## Results

### Socio-demographic characteristics of respondent

In this study, out of 626 eligible mothers of 6–23 months aged children 611 (97.6 %) were provided the data. Briefly, of 611 mothers 224 (36.7 %) were in the age group of 25–29 followed by age group < = 24 years (21.3 %) with mean age of 27.54 years. Regarding marital status of respondents, almost all (96 %). Respondents’ educational status showed that most (42 %) of them were primary school followed by illiterate ones (25 %). Most (465, 76.1 %) of participants were housewife, and 358 (58.6 %) of husbands’ occupation was farmer (Table 1).

**Table 1:** Sociodemographic characteristics of respondents, Arsi Negele, Southern Ethiopia, 2015

Characteristics	Frequency	Percent
Age of mothers/caretakers		
< =24 years	173	28.3
25–29 years	224	36.7
30–34 years	130	21.3
> = 35 years	84	13.7
Mean age:		27.54
Educational status of mother/caretaker		
Illiterate	151	24.7
Read and write only	86	14.1
Primary school	256	41.9
Secondary school	105	17.2
College/university	13	2.1
Mothers/caretakers occupation		
Housewife	465	76.1
Farmer	69	11.3
Housemaid	33	5.4
Merchant	30	4.9
Daily laborer	3	0.5
Others	11	1.8
Educational status of husband		
Illiterate	67	11.0
Read and write only	59	9.7
Primary school	233	38.1
Secondary school	215	35.2
College/university	37	6.1
Husband’s occupation		
Farmer	358	58.6
Private employee	158	25.9
Government employee	31	5.1
Merchant	28	4.6
Daily laborer	23	3.8
Others	13	2.1
Family income per month		
< =999 ETB	289	47.3
1000–1999 ETB	265	43.4
2000–2999 ETB	28	4.6
3000–3999 ETB	12	2.0
> =4000 ETB	17	2.8
Ethnicity		
Oromo	504	82.5
Kanbata	59	9.7
Others	48	7.9
Family size		
1–3	109	17.8
4–6	347	56.8
> =7	155	25.4

### Maternal healthcare related variables

Maternal characteristics and healthcare utilization was assessed. Accordingly, more than half (56.8 %) of the respondents were multipara (2–5 births), about two third of them gave birth at home and majority (90.5 % and 88 %) of mothers were attended ANC and PNC respectively (Table 2).

**Table 2:** Maternal obstetric related characteristics Arsi Negele, Southern Ethiopia, 2015

Characteristics	Frequency	Percent
Parity of mothers		
Primiparous (1)	106	17.3
Multiparous (2–4)	347	56.8
Grand multipara (5+)	158	25.9
Place of delivery		
Home	385	63.0
Public hospital	119	19.5
Primary health center	93	15.2
Private clinic	14	2.3
Attend ANC		
Yes	553	90.5
No	58	9.5
Attend PNC		
Yes	541	88.5
No	70	11.5
Attended Health Development Army		
Yes	451	73.8
No	160	26.2
Radio possession		
Yes	364	59.6
No	247	40.4

### Child characteristics, feeding practices and dietary assessment

Table 3 presents child characteristics such as age, gender, feeding practice of mothers/caregivers and dietary assessment using 24 h recall. Of 611 children enrolled in the study 331 (54 %) were males and 280 (46 %) were females. of 611 children 43 % of them were 18–23 months of age, 32 % were aged 12–17 months and the rest quarter were aged 6–11 months with mean age of 18.7 months. The commonest (95 %) dietary recall was grain, roots and tubers that 95.4 % while, the least (11 %) one was flesh foods.

**Table 3:** Feeding practice of mothers for their children, Arsi Negele, Southern Ethiopia, 2014/15

Characteristics	Frequency	Percent
Sex of the child		
Male	331	54.2
Female	280	45.8
Age of the child		
6–11 months	152	24.9
12–17 months	196	32.1
18–23 months	263	43.0
Mean		18.7
Ever breast fed your child		
Yes	605	99.0
No	6	1.0
Still breast feeding your child?		
Yes	500	81.8
No	111	18.2
Age at which the child stop breast feeding		
< 6 months	6	5.4
6–12 months	3	2.7
> 12 months	102	91.9
Ever started complementary feeding for your child		
Yes	610	99.8
No	1	0.2
When you started complementary feeding for your child		
Less than 6 months	82	13.4
At 6 months	443	72.5
Greater than 6 months	86	14.1
Did you use separate container to feed your child?		
Yes	586	95.9
No	24	4.1
Type of separate container use		
Bottle	201	32.9
Cup with spoon	311	50.9
Others (specify)	99	16.2
Number of times you fed your child per day		
Once only	31	5.1
2–3 times	211	34.5
3–4 times	311	50.9
4+ times	58	9.5
Did you include snacks between foods		
Yes	461	75.5
No	149	24.5
Type of food or fluid mostly provided to your child?		
Porridge	334	56.1
Adult types	139	22.7
Gruel	226	37.0
Dietary assessment using 24 h recall		
Grain, roots and tubers	583	95.4
Legumes and nuts	172	28.2
Diary product	320	52.4
Flesh foods	69	11.3
Eggs	136	22.3
VA rich fruits and vegetables	171	28.0
Other rich fruits and vegetables	107	17.5

### Indicators for Complementary feeding

Indicators of complementary feeding was assessed and only 115 (19 %) mothers offered four or more food groups to their child meeting the minimum dietary diversity criteria on the day preceding the study. Majority (72.5 %) of the mothers initiated complementary feeding at 6 months (timely initiated complementary feeding). About two third (67.3 %) mothers fed their children more than two times a day, the day preceding the survey. From the three combining indicators, overall prevalence of appropriate complementary feeding practices was 9.5 % (95 % CI = 7.0-12.0, 57/611). The minimum acceptable diet of the studied children was 12.3 % (75/611).

### Factors associated with Complementary Feeding Practices, bivariate and multivariable analyses

Table 4 shows factors associated with appropriate complementary feeding practices of children aged 6–23 months: a bivariate and multivariate analyses. Variables having *P.value* less than 0.1 in bivariate analyses were re-entered in to binary logistic regression to control for possible potential confounders. These variables were maternal education, occupation and parity, family size, household income, child age, place of delivery and maternal ANC, PNC and HDAs follow up. Accordingly, of total entered variables only maternal education, family size and child age were found to be associated with appropriate complementary feeding while the rest variables were not associated or lost association after controlling for potential confounders though associated in bivariate analyses. Maternal education found to be statistically significantly associated with complementary feeding that those mothers who were secondary school and above and primary school were about three times more likely to practice appropriate complementary feeding than those who had no formal education, AOR = 3.24 and 3.21, respectively. Another predictor’s variable showing association was family size that those households’ having 1–3 persons were more likely to practice appropriate complementary feeding compared with those households’ having > =7 family (AOR = 12.37, 95 % CI: 1.10–139.7) while, family size of 4–6 persons lost association after adjustment. Finally, the odds of appropriate complementary feeding practices among older children (12–17 months and 18–23 months) was nearly three times compared with younger (6–11 months) ones with AOR = 2.75 (95%CI: 1.07–7.03); 2.64 (95 % CI: 1.06–6.74) respectively.

**Table 4:** Factors associated with complementary feeding of child age 6–23 months, Arsi Negele, Southern Ethiopia, 2015

Variables	Complementary feeding practices			
	Appropriate	Inappropriate	COR (95 % CI)	AOR (95 % CI)
	No (%)	No (%)		
Maternal education				
No formal education	10 (17.5)	228 (41.2)	1.00	1.00
Primary school	29 (50.9)	227 (41.0)	2.91 (1.39–611)	3.24 (1.28–8.20)
Secondary school and above	18 (30.6)	99 (17.9)	4.14 (1.85–9.30)	3.21 (1.1.07–9.70)
Maternal occupation				
Housewife	37 (64.9)	428 (77.3)	1.00	1.00
Private business	13 (22.8)	64 (11.6)	2.35 (1.18–4.66)	1.66 (0.76–3.64)
Farmers	7 (12.3)	62 (11.2)	1.30 (0.56–3.06)	1.14 (0.46–2.87)
Family size				
1–3	11 (19.3)	98 (17.7)	2.37 (0.89–6.33)	12.37 (1.10–139.7)
4–6	39 (68.4)	308 (55.6)	2.68 (1.17–6.13)	1.18 (0.2–7.07)
> = 7	7 (12.3)	148 (26.7)	1.00	1.00
Family income/month				
< = 999	18 (6.2)	271 (93.8)	0.50 (0.11–2.35)	0.53 (0.10–2.83)
1000–1999 ETB	30 (11.3)	235 (88.3)	0.96 (0.21–4.39)	1.03(0.20–5.32)
2000–2999 ETB	5 (17.9)	23 (82.1)	1.63 (0.28–9.52)	1.71 (0.30–12.10)
3000–3999 ETB	2 (16.7)	10 (83.3)	1.50 (0.18–12.46)	1.80 (0.12–24.85)
4000+ ETB	2 (16.7)	15 (88.2)	1.00	1.00
Age of the child				
6–11 months	7 (12.3)	145 (26.2)	1.00	1.00
12–17 months	22 (38.6)	174 (31.4)	2.62 (1.09–6.60)	2.75 (1.07–7.03)**
18–23 months	28 (49.1)	235 (42.4)	2.47 (1.05–5.80)	2.67 (1.06–6.74)**
Place of delivery				
Health facility	28 (12.4)	198 (87.6)	1.74 (1.00–3.00)	1.37 (0.74–2.55)
Home	29 (7.5)	356 (92.5)	1.00	1.00
Attended ANC				
Yes	56 (98.2)	494 (89.2)	6.80 (0.92–50.03)	1.65 (0.11–24.85)
No	1 (1.8)	60 (10.8)	1.00	1.00
Attended PNC				
Yes	56 (98.2)	485 (87.5)	7.96 (1.08–58.48)	1.86 (0.79–4.36)
No	1(1.8)	69 (12.5)	1.00	1.00
Attended HDAs (1–5)				
Yes	49 (86.0)	402 (72.6)	1.32 (1.07–5.00)	1.61 (0.70–3.68)
No	8 (14.0)	152 (27.4)	1.00	1.00
Parity of the mother				
Primiparous (1)	9 (15.8)	97 (17.5)	2.00 (0.72–5.55)	0.12 (0.01–1.44)
Multipara (2–4)	41 (71.9)	306 (55.2)	2.89 (1.27–6.59)	1.31 (0.21–8.08)
Grand multipara (5+)	7 (12.3	151 (27.3)	1.00	1.00

*COR* Crude odds ratio, *AOR* Adjusted odds ratio, *CI* Confidence interval

* *P* < 0.05, ** *P* < 0.01

## Discussions

A study conducted on prevalence of appropriate complementary feeding practices and associated factors had a response rate of 97.6 % and identified the overall prevalence of appropriate complementary feeding practices as 9.5 % (95 % CI:7.0–12.0, 57/611). Minimum acceptable diet in this study is 12.3 %; minimum dietary diversity is 18.8 %; minimum feeding frequency is 67.3 % and timely initiation of complementary feeding at 6 months is 72.5 %. Maternal education, household family size and child’s age are the variables found to be associated with appropriate complementary feeding practices among children aged 6–23 months.

In this study appropriate complementary feeding practices is very low compared with other similar studies conducted in five Asian counties and Tanzania reporting higher figures [9, 15]. This might be due to poor SES observed in the current study that results in low accessibility to food and high illiteracy rate compared to other study sites. However, the current findings is relatively higher to the National figures (5 %) (9). the reasons for the discrepancy might be our study site being adjacent to urban areas while, the National (EDHS, 2011) encompasses different communities, mainly from rural with various complementary feeding practices. This is the first study assessed appropriate complementary feeding practices among mothers of children aged 6–23 months using a three combined indicators in the study area. Therefore, our findings has an implication for improving the practices of appropriate complementary feeding as per recommendation by WHO that influencing appropriate feeding practices is as critical as influencing availability and use of adequate foods (8). In addition, the Ethiopian National strategy for IYCF and WHO recommends an accurate information and skilled support from the family, community and health system to scale up the optimal complementary feeding practices to children age 6–23 months (5, 8).

The prevalence of minimum acceptable diet in this study is only 12.3 %. This is almost coincides with the findings of India [12] but higher to the national prevalence (4.2 %) report of EDHS, 2011 [4]. A relatively higher findings observed in our study may be due to educational differences in that relatively lower illiteracy rate observed in this study and being adjacent to urban area where there is an access to better maternal and child healthcare, high antenatal follow up.

However, this finding is lower to other similar studies conducted in Sri Lanka (68 %), Bangladesh (40 %) and Nepal (32 %), [10, 11, 13]. This might be associated with poor socioeconomic status observed in the current study that most of the caregivers earn lower monthly salary (<=50USD) and low literacy rate of mothers compared with other studies reported higher minimum acceptable diet prevalence.

The prevalence of minimum dietary diversity in the current study was 18.8 %. Our finding is consistent with the Nigerian study of 10 years trend analysis of complementary feeding indicators (26). However, the current findings is lower to other similar studies elsewhere reporting minimum dietary frequency of 38–71 % [10, 11, 13, 15]. This might be due to the fact that there are educational, socioeconomic and cultural differences.

However, this finding is higher to similar studies conducted in other parts of Ethiopia reporting 8.5–10.8 % [21, 23]. This is due to the fact that the current study conducted in semi-urban area where there is better access to healthcare services than other studies and better maternal literacy than other studies in the current study area.

The current study determined that the minimum feeding frequency was 67.3 %. Nevertheless, this finding is lower to studies conducted in Sri Lanka (88.3 %), Bangladesh (81 %), Nepal (82 %), coastal South India (77.5 %) and Derashe, Southern Ethiopia (95 %) [10, 11, 13, 20, 27]. This might be as a result of social, cultural and educational differences existed between the current study and others.

This is however, higher to national prevalence of 51 % (9) and other studies conducted elsewhere reporting minimum feeding frequency of 18–61 % [13, 16, 18, 22, 25, 28]. Higher minimum feeding frequency figure observed in this study as compared to the National figure (51 %) might be because EDHS, 2011 was a nationally representative survey with a wide range of child feeding styles in different regions of Ethiopia. Furthermore, the higher figure observed in our study may be due to current expansion of HEWs in the study area that focused on antenatal, postnatal and child care education which in turn increases maternal exposure to healthcare workers so that increases their practices.

In the current study about 72.5 % of mothers/caretakers started complementary feeding at 6 months of age of their child which is similar with Bangladesh and Nepal studies reported 70–71 % [11, 13]. However, about three-forth prevalence we detected is higher than the national prevalence (51 %) (4). This figure is still lower to WHO recommendation of more than 80 % of 6–8 months children should initiate complementary feeding at 6 months of age [26].

However, in the current findings, the correct time of introduction of complementary feeding is better than other similar studies conducted elsewhere [12, 22, 23, 29, 30]. Healthcare access such as antenatal care, postnatal care and institutional delivery were better in the current study area so that better awareness and practices on correct time of complementary feeding introduction compared with other studies could be the reasons for the discrepancy.

Of factors associated with appropriate complementary feeding practice, maternal education shows strong association that mothers who are at primary and secondary schools and above are more likely to practice appropriate complementary feeding compared with those mothers who have no formal education which is consistent with similar studies conducted elsewhere [9, 12, 17, 18, 24, 25]. To improve complementary feeding practices, there is a need to target the communities with low level of maternal education. In addition, there is strong evidences that maternal education is associated with improved child-care practices related to health and nutrition and reduced odds of stunting, and better ability to access and benefit from interventions [1]. But, there is a need to conduct a further follow up study to validate our findings.

Child age is also found to be predictor variables as older children (12–17 and 18–23 months) are about three times more likely to feed appropriately compared with younger children (6–11 months). Similarly, studies conducted in five Asian countries and Tanzania, and Northern part of Ethiopia reported child age as a predictor variable [9, 15, 28]. This might give an opportunities for the health progamme planners to pay more attention to the feeding of younger children. Because of the fact that, the problem of appropriate feeding of 6–8 months age children is supported by the findings from previous study conducted in South Ethiopia, a neighboring woreda to our study area, showed high prevalence of stunting (43 %) among 6–8 months children, there is a need to give more emphasis to feeding of 6–8 months children [22].

Another important determinant factor associated with appropriate complementary feeding practices is family size in that those mothers having lower family size (1–3 persons/head) are more practicing it as compared to those mothers having higher family size (> = 7 persons/head). This may be due to inadequacy of food (insecurity) in those households having more family size; and mothers having too little time to prepare food or to feed their children. However, our findings should be supported by community based follow up study to elicit the true association between the variables.

Similarly, maternal occupation, postnatal care follow up, place of delivery and maternal parity were all associated with complementary feeding practices in bivariate analyses but lost associations after adjusted for potential confounders.

On the other hand, this study revealed that there is no association between antenatal care visit and appropriate complementary feeding practice and it disagrees with the studies conducted in Nigeria and five Asian countries [9, 25] where inadequate antenatal care was associated with inappropriate complementary feeding. This might be due to more attention of healthcare workers on pregnancy and related factor during antenatal visit rather than child feeding practices in the current setting.

Our study is not free from limitations. The study being cross sectional, temporality is a problem that we cannot ascribe the causality to those factors found to be associated with appropriate complementary feeding. Recall and social desirability bias may be introduced as frequency, types of foods and time of initial depends on respondents own memory. The 24 h dietary diversity recall may show only the current feeding and needs repeated measures. Another limitation of our study may be generalizability, as we sampled only the population from a single woreda that may not be representative of the region.

## Conclusions

The prevalence of timely initiation of complementary feeding at 6 months, minimum dietary diversity, minimum feeding frequency and minimum acceptable diet were low. The overall prevalence of appropriate complementary feeding practices was also very low which have impact on the health of infants and young children and indicated the importance of immediate action to promote appropriate complementary feeding. Educated mothers, older children aged 12–23 months and smaller family size were factors that can increase appropriate complementary feeding practice.

The need to establish and strengthen inter-sectoral collaboration to think over the possibilities of increasing appropriate complementary feeding practices based on the three indicators. What is needed is to scale up these successful interventions to levels that would make an impact. Finally, quality counselling of mothers and caregivers, and appropriate behavioral change communication to other family and community decision-makers, are essential for improving infant and young child feeding practices with special emphases given to poorly educated mothers, younger children (6–8 months) and households with high number of families.

## Abbreviations

ANC: Ante natal care
AOR: Adjusted odds ratio
CI: Confidence interval
COR: Crude odds ratio
EDHS: Ethiopian Demographic and Health Survey
WHO: World Health Organization
